# Binocular Onset Rivalry at the Time of Saccades and Stimulus Jumps

**DOI:** 10.1371/journal.pone.0020017

**Published:** 2011-06-15

**Authors:** Joke P. Kalisvaart, Sumientra M. Rampersad, Jeroen Goossens

**Affiliations:** Radboud University Nijmegen Medical Centre, Donders Institute for Brain, Cognition and Behaviour, Nijmegen, The Netherlands; University of Southern California, United States of America

## Abstract

Recent studies suggest that binocular rivalry at stimulus onset, so called onset rivalry, differs from rivalry during sustained viewing. These observations raise the interesting question whether there is a relation between onset rivalry and rivalry in the presence of eye movements. We therefore studied binocular rivalry when stimuli jumped from one visual hemifield to the other, either through a saccade or through a passive stimulus displacement, and we compared rivalry after such displacements with onset and sustained rivalry. We presented opponent motion, orthogonal gratings and face/house stimuli through a stereoscope. For all three stimulus types we found that subjects showed a strong preference for stimuli in one eye or one hemifield (Experiment 1), and that these subject-specific biases did not persist during sustained viewing (Experiment 2). These results confirm and extend previous findings obtained with gratings. The results from the main experiment (Experiment 3) showed that after a passive stimulus jump, switching probability was low when the preferred eye was dominant before a stimulus jump, but when the non-preferred eye was dominant beforehand, switching probability was comparatively high. The results thus showed that dominance after a stimulus jump was tightly related to eye dominance at stimulus onset. In the saccade condition, however, these subject-specific biases were systematically reduced, indicating that the influence of saccades can be understood from a systematic attenuation of the subjects' onset rivalry biases. Taken together, our findings demonstrate a relation between onset rivalry and rivalry after retinal shifts and involvement of extra-retinal signals in binocular rivalry.

## Introduction

When both eyes are presented with distinctly different images, a phenomenon called binocular rivalry arises. Instead of merging the images of both eyes into a single binocular percept, the two images are perceived alternately in a quasi-regular fashion (for reviews, see [1], [2], [3], [4]). This bistable behavior has attracted considerable attention, partly because it provides a clear dissociation between stimulus and visual awareness. Thus far, however, the neural mechanisms underlying binocular rivalry remain poorly understood.

Several studies have used brief or intermittent stimulus presentation with varying interstimulus intervals [5], [6], [7], [8], [9], [10]. These studies revealed that perception becomes remarkably stable when short stimulus presentations (0.5–1.2 sec) are combined with relatively long interstimulus intervals (>1 s), whereas short interstimulus intervals (<0.5 s) promote percept alternations with every new stimulus presentation. The observed perceptual stabilization was assumed to reflect temporary suppression or slowing of the physiological processes underlying binocular rivalry. Recent findings suggest, however, that rivalry at the beginning of a trial, so called onset rivalry, may be different from sustained rivalry [11], [12], [13], [14]. For example, Mamassian & Goutcher [11] found that contrast differences between the two stimuli cause a strong eye bias at stimulus onset that wears off during the course of the trial toward a more equal dominance of the two eyes. Furthermore, Carter & Cavanagh [13] showed that this onset bias also occurs with equiluminant stimuli but that it is highly specific to certain locations in the visual field and that these locations differ between subjects. These findings raise the interesting question whether there is a relation between onset rivalry and rivalry in the presence of eye movements. Because eye movements interrupt stimulus viewing for the duration of the saccade and in addition cause the retinal images to change, the implications of saccades for models of binocular rivalry are far from trivial.

In current models, binocular rivalry typically revolves around two mechanisms. Mutual inhibition between monocular cell populations, which induces suppression of one percept while the other is dominant, and slow self-adaptation of cells within each population, which causes the dominant percept to be replaced by the other percept after a certain period. In line with these models, rivalry has been found to slow down if the stimulus is moving, preventing adaptation [15]. It is implicitly assumed, however, that these mechanisms act locally, affecting only populations of cells in retinotopic visual areas that have their receptive fields at the location of the stimulus. In agreement with this assumption, adaptation studies found that, at least for lower order stimuli such as gratings, adaptation only occurs in retinotopically matched locations [16], [17] although the strength of the aftereffect is found to be gaze-dependent [18].

When a saccade causes a displacement of the visual stimulus across the retina, however, e.g., from one hemifield to the other, a new population of cells will be stimulated. Clearly, the cells in this new population will have a different adaptation state than the ones stimulated before the movement because they have experienced a different visual history. These models therefore predict that a rivaling stimulus that has been shifted to a new retinal location is processed as a new stimulus and that it should make no difference whether the retinal image shift is caused by a physical displacement of the stimulus or by a saccadic eye movement. Alternatively, the balance between excitation and inhibition could be under more direct, active neural control. For example, saccades might help maintaining perceptual continuity, as suggested by Ross & Ma-Wyatt [19], and cause less perceptual switches than passive stimulus jumps.

To test these different possibilities, we first characterized onset rivalry and rivalry during sustained viewing in two separate control experiments (Experiment 1 and 2). We then studied state changes in binocular rivalry when retinal image shifts were produced actively by saccades or passively by displacements of the stimulus on the projection screen (Experiment 3), and we compared rivalry after such retinal displacements to the behavior observed at stimulus onset and during sustained viewing. Apart from the commonly used grating stimulus, we also used a face/house stimulus and a motion stimulus because these different stimulus types involve at least partly different (dorsal and ventral) visual pathways in the brain.

We report that both active and passive retinal displacements produced subject-specific eye/hemifield preferences that were very similar to those seen at stimulus onset. Our data thus suggests that these retinal image shifts trigger onset rivalry. Interestingly, however, the behavior observed after saccades versus stimulus jumps was not the same; saccades produced a significant attenuation of the eye/hemifield preferences. We conclude therefore that non-visual, oculomotor signals have a significant impact on the rivalry process. This implies that state changes in binocular rivalry are not only determined by passive adaptation processes, but also involve active neural control components.

## Methods

### Subjects

Eleven adult human subjects participated in the experiments. All subjects had normal, or corrected to normal, visual acuity. Subjects JK, SR and JG had previous experience with the tasks. The other subjects were inexperienced and naive as to the purpose of the investigation.

The volunteers were informed about the experimental procedures and gave informed consent in writing before the start of the experiments. All procedures were in accordance with the Declaration of Helsinki, and approved by the Radboud University Medical Centre. Table 1 lists age and gender of each participant.

**Table 1:** Subject characteristics.

Subject	Gender(M/F)	Age(Years)	Stimulus type
DB	F	24	MFG
FW	M	23	MF
JB	F	25	MF
JG	M	39	MG
JK	F	25	MFG
JT	M	24	MF
JV	F	33	F
MV	F	28	MF
RH	M	26	F
SR	F	23	MFG
TG	M	30	MFG

The column labeled ‘stimulus type’ indicates which stimulus types were tested in each subject: M(otion), F(ace/house) and/or G(ratings).

### Setup

**Figure 1:**
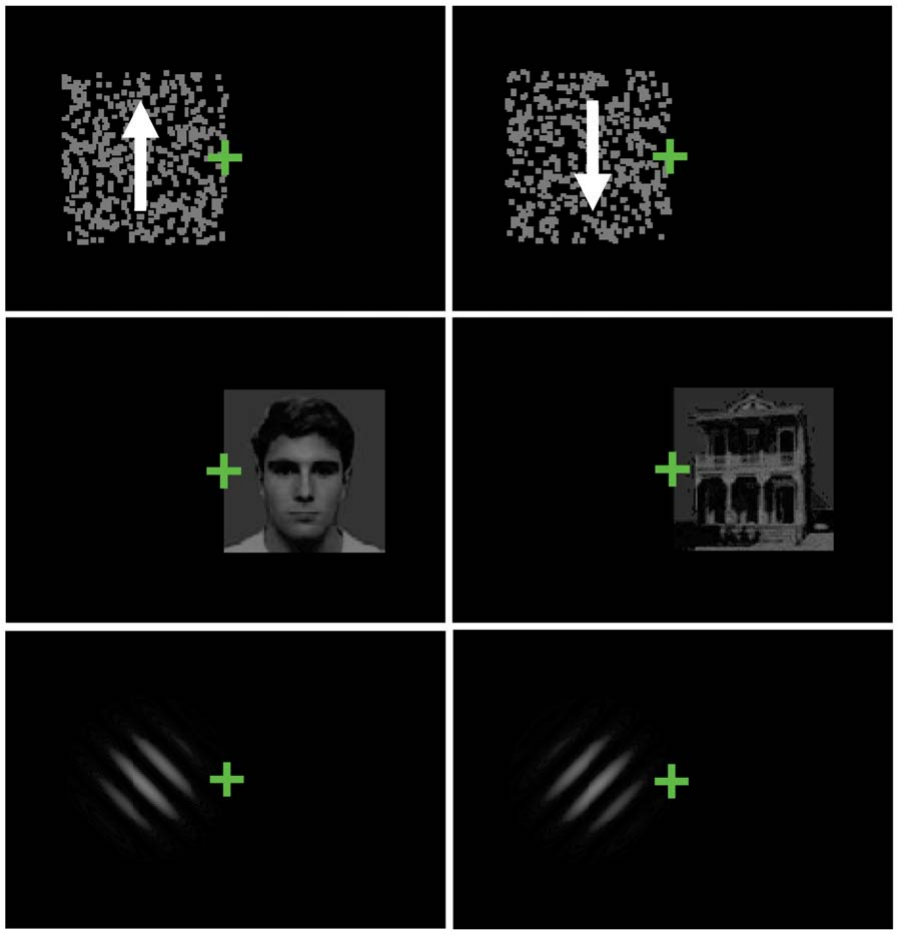
Illustration of the different stimuli used in this study. Top: opponent motion stimulus, middle: face/house stimulus, bottom: oblique grating stimulus. The arrows indicating movement direction in the motion stimulus were not present in the real stimulus. Each panel shows the stimulus as it was presented to one eye. The stimulus was always positioned directly to the left (as shown for the motion and the grating stimuli) or to the right (as shown for the face/house stimulus) of the fixation point.

Subjects were seated in a dark room at 52 cm from a projection screen on which visual stimuli were back projected with an LCD projector (Panasonic PT-AX100E) at 60 Hz. The total size of the projection area was 57×32 cm with a resolution of 1280×720 pixels. The subject watched the screen through a front-mirror stereoscope (HyperView, Berezin, U.S.). Head movements were restricted with a chin support or with a bite board. Dichoptic stimuli (Figure 1) were generated with Matlab (The MathWorks, Inc.) using the Psychophysics Toolbox extensions [20], [21]. It was either a 4×4° random dot kinematogram with dots moving coherently in opposite directions, a 4×4° face/house stimulus (modified after [22]) or a circular sinusoidal grating with a diameter of 4°. The motion stimulus consisted of 500 dots (0.14° white squares) moving vertically with a speed of 2.75° per second (1 pixel/frame). Dots had asynchronous lifetimes of 0.33 s. When a dot died, it was redrawn at a random position within the stimulus area. The spatial frequency of the grating was 1 cycle per degree. Stimulus contrast was the same for images presented to the left and right eye, with a maximal luminance of 98 cd/m^2^ and a background luminance of 1.3 cd/m^2^ (Minolta LS-100 Luminance meter).

Each trial started with a 0.46° fixation cross, and after a fixed delay of 1 s, a rivalrous stimulus was presented. The stimuli were always presented directly left or right from the fixation point, such that the center of the stimulus was located at a retinal eccentricity of 2°. Motion, face/house and grating stimuli were tested in separate sessions. When the left eye watched the upward motion, the face, or the left-oblique grating, the right eye watched the downward motion, the house, or the right-oblique grating and vice versa. The hemifield in which the stimulus was presented also varied between trials. This resulted in the four possible stimulus configurations listed in Table 2 (for the motion stimulus), which were presented in pseudorandom order.

**Table 2:** Four possible configuration of the motion stimulus.

	Visual hemifield containing stimulus	Image presented to left eye	Image presented to right eye
1	Left	Up	Down
2	Left	Down	Up
3	Right	Up	Down
4	Right	Down	Up

For the face/house stimulus, up and down were replaced by face and house respectively. For the grating stimulus, up and down were replaced by left- and right-oblique, respectively.

Subjects indicated their percept by pressing one of two mouse buttons. Button presses were recorded by the stimulus program with a temporal resolution of 60 Hz.

In part of the experiments we measured eye movements in two dimensions with either an infrared eye tracker (Eyelink® II, Version 1.11, SR Reasearch, Canada; subjects DB, JG, JK and JT) or with the scleral search coil method [23] (subjects JG and DB). The spatial resolution of the eye tracker was in the order of 0.5 degrees (root mean square measure). The spatial resolution of the search coil system was better than 0.3 minutes of arc. The results of these control measurements indicated that gaze remained centered on the fixation point when required, with saccade amplitudes during fixation <0.2°.

### Onset and sustained rivalry

We first characterized onset rivalry and rivalry during sustained viewing in two separate experiments. In Experiment 1 we asked subjects to indicate their first dominant percept (e.g., upward or downward motion) after stimulus onset. The stimulus remained present until the subject responded (typically within 400–900 ms). Each subject completed 100 trials per stimulus type, 50 in each hemifield. In Experiment 2, the peripheral stimuli were presented for 30 seconds, and subjects were asked to continuously indicate their dominant percept while fixating the straight-ahead fixation point. Stimuli were presented to the left and to the right of the fixation point as in Experiment 1. Each subject completed 48 (JB, JV and RH) or 80 trials (all other subjects) per stimulus type, balanced between eyes and hemifields (c.f., Table 2) in a pseudorandom order.

## Results

### Experiment 1: Initial percept

Experiments by Carter and Cavanagh [13] have indicated that rivalrous grating stimuli produce subject-specific onset biases. The aim of the first experiment was to quantify these biases for the observers that took part in the present study, and to test whether they exist also for other types of stimuli. Towards that end, we presented peripheral stimuli while subjects maintained straight-ahead fixation and we asked them to indicate their first dominant percept after stimulus onset (Methods).

**Figure 2:**
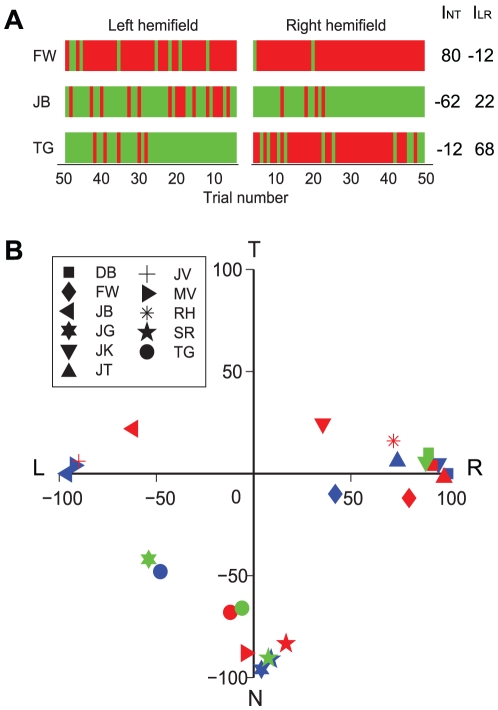
Subject-specific onset preference. A: Data from three typical subjects (face/house stimulus). Each colored vertical line shows the onset response in a single trial: right eye or left eye dominance is indicated in red and green, respectively. Trials in which the stimulus was presented in the left or the right hemifield are presented on the left-hand and right-hand side of the center, respectively. The first trial is shown in the center, trial number increases outwards. Values marked *I_LR_* and *I_NT_* at the right hand side of the figure show preference indices (see text). B: Indices quantifying the left/right and nasal/temporal preference at trial onset. *I_NT_* is plotted as a function of *I_LR_* for each subject. Colors indicate different stimulus types. Blue: motion stimulus (9 subjects), red: face/house stimulus (10 subjects), green: grating stimulus (5 subjects).

Figure 2A shows the responses from three representative subjects when (face/house) stimuli were presented in either the left hemifield (left column) or right hemifield (right column). Red and green vertical lines identify onset dominance of the stimulus presented in the right and left eye, respectively. Note that these subjects showed one of three characteristic response patterns: FW almost always showed right eye dominance at stimulus onset, whereas subject JB showed an onset preference for the left eye, regardless whether the stimulus was located in the left or the right hemifield. For subject TG, however, there was a bias that depended on the retinal location of the stimulus. This subject showed a strong preference for the left eye when the stimulus was located in the left visual hemifield and a strong preference for the right eye when the stimulus was located in the right visual hemifield. The results thus indicate a significant preference in this subject for perceiving the image in the nasal part of the retina over perceiving the image in the temporal part at stimulus onset.

To quantify this behavior for each subject we calculated an index *I_LR_* that measured the preference for the left versus the right eye, and an index *I_NT_* that measured the preference for nasal versus temporal retinal halves. The indices were defined in the following way:
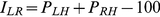
(1)

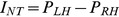
(2)where *P_LH_* and *P_RH_* are the percentage of trials in which the right eye was dominant at the beginning of a trial while the stimulus was presented in the left or the right hemifield, respectively. The values thus range from −100 for complete left/nasal preference to +100 for complete right/temporal preference. Figure 2A lists the values of *I_RL_* and *I_NT_* for the three example subjects.

The scatter plot in Figure 2B shows *I_NT_* as a function of *I_LR_* for all eleven subjects (different symbols) for the three different stimulus types (different colors). Note that the data from most subjects are located near the end of either the horizontal or vertical axis, indicating a strong preference for one eye or one hemiretina, respectively. In our group of subjects, we found strong preferences for the right eye, the left eye and the nasal retina, but not for the temporal retina. This behavior was consistent across stimulus types in the sense that each subject showed a qualitatively similar onset bias for each stimulus type. Pearson's correlations between the motion, the face/house stimulus, and the grating stimulus ranged between 0.84 and 0.86 for the *I_RL_* index and between 0.64 and 0.98 for the *I_NT_* index. The magnitude of the onset biases, however, was not identical. Most individuals did show significantly different onset biases for face/house, grating and motion stimuli (Fisher exact tests, p<0.05), but these differences were typically small, and showed no consistent pattern across subjects.

### Experiment 2: Sustained rivalry

Previous studies have reported that the percept biases at stimulus onset do not persist during sustained viewing [11], [13]. The objective of the second experiment was to verify this behavior for our group of subjects and to estimate how fast the onset effect wears off for the different stimulus types that we have used. Towards that end, subjects were required to fixate straight-ahead during 30 second trials, and continuously indicate their dominant percept of the peripheral stimulus by pressing one of two mouse buttons (Methods).

**Figure 3:**
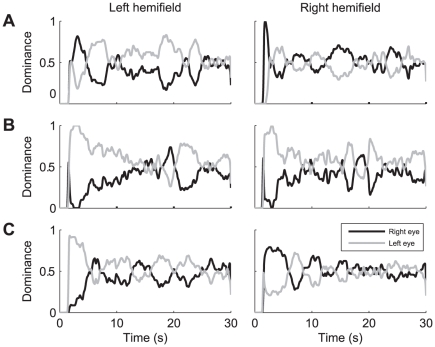
Average eye dominance as function of time in the sustained rivalry motion task. A: subject FW. B: subject JB. C: subject TG. Right- and left-hand panels show data from trials in which the stimulus was in the right or the left hemifield, respectively. Black line: right eye. Gray line: left eye.

Figure 3 shows for three subjects (FW, JB and TG) the probability of right and left eye dominance as function of time during motion trials. Data in the left- and right-hand panels are averaged across all trials in the left and right hemifield, respectively. Note that, after the initial reaction time in which neither button was pressed, there was first a strong bias for images in either the left or the right eye. This bias was consistent with the bias observed in Experiment 1 (c.f., Figure 2A): subjects FW and JB again showed an onset preference for images in the right (Figure 3A) and the left eye (Figure 3B), respectively, whereas subject TG again showed a right-eye preference if stimuli were presented in the right hemifield but a left-eye preference if stimuli were shown in the left hemifield (Figure 3C). Accordingly, the onset preferences as measured in Experiment 2 were strongly correlated with the onset preferences measured in Experiment 1 (Pearson's correlation across subjects and stimulus types: r = 0.85, p<0.001). After the strong initial bias for either left or right eye dominance, however, the instantaneous probabilities for left and right eye dominance both converged on an average value of about 0.5. The data thus indicate that rivalry entered a steady state in which both eyes were dominant for approximately 50% of the time. In all our subjects, this steady state was reached within the first 10 seconds of the trials.

**Figure 4:**
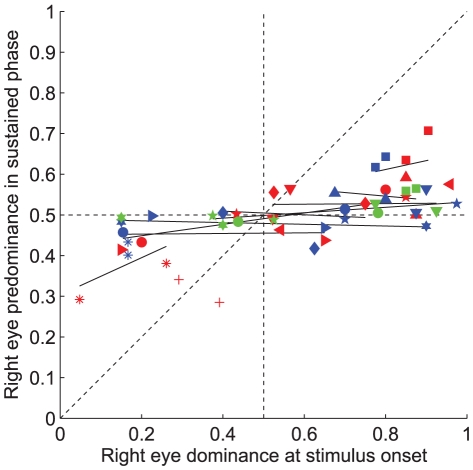
Eye predominance during sustained rivalry phase as function of eye dominance at stimulus onset. Right eye predominance in the sustained rivalry phase (Experiment 2) is plotted as function of right eye dominance probability at stimulus onset. Symbols identify the same subjects as in Figure 3. Blue: motion stimulus (9 subjects), red: face/house (10 subjects) stimulus, green: grating stimulus (5 subjects). Black lines: linear regression lines for 9 subjects that completed the experiment with more than one stimulus type.

Figure 4 compares the percentage of right eye dominance at stimulus onset as observed in Experiment 2 with the right eye predominance during sustained viewing. The right eye predominance during sustained viewing was calculated as the total proportion of time that the right eye was dominant during all but the first two dominance states in each trial (i.e., excluding the first dominant state for both the left and the right eye). This was done separately for stimuli in the left and right hemifield and for each stimulus type, resulting in 2, 4 or 6 data points per subject (depending on the number of stimulus types tested). For each of the nine subjects that completed the experiments with at least two different stimulus types (c.f., Table 1), we then calculated linear regression coefficients and plotted the resulting regression lines. If onset rivalry and sustained rivalry result from the same process, the slopes are expected to be around 1.0. However, in line with previous results by Carter & Cavanagh [13] we found that for all nine subjects the slopes of the regression lines were small, and significantly less than 1 (F-test, p<0.03). With a mean (±SD) slope of the linear regression lines of 0.08±0.17 across subjects, our data strongly support the notion that sustained rivalry and onset rivalry are independent. Pearson correlation coefficients were indeed not statistically significant in eight of nine subjects (t-tests, p>0.05).

Note, in Figure 4, that for the majority of our subjects, the average eye dominance in the sustained phase was close to 50% whereas their eye/hemifield preference at trial onset was typically biased. Only two subjects (DB and JV) showed a significant predominance of one eye over the other in the sustained phase (Wilcoxon rank sum test, p<0.05), but that difference in dominance was not as extreme as at stimulus onset.

### Experiment 3: Retinal image shifts

#### Methods

**Figure 5:**
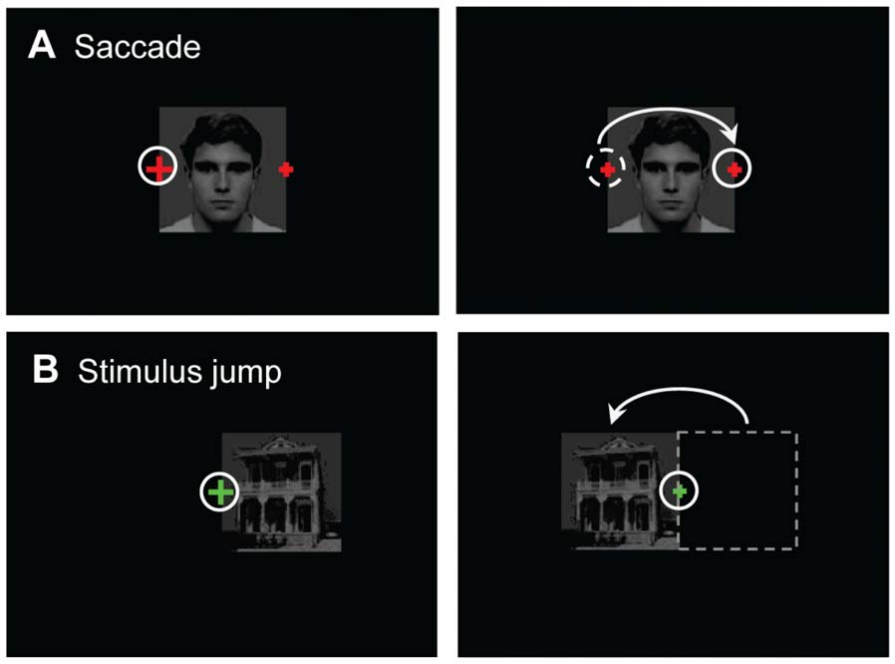
Illustration of the two trial types in Experiment 3. White circles indicate the subject's gaze position. Arrows denote a saccade or stimulus jump. Each panel shows the input for one eye only. A: Saccade trials. The subjects looked at the large, red cross until it shrunk. Then the subject made a saccade to the other cross, thus actively changing the retinal input. B: Stimulus jump trials. The subject looked at the green fixation cross at the center of the screen and kept fixation there during the whole trial. After a certain delay, the stimulus jumped to the opposite side, resulting in the same retinal displacement as in A, but this time the displacement was passive.

In Experiment 3, we quantified percept state changes at the time of a retinal image shift. In this paradigm, subjects watched the stimulus and during the sustained rivalry phase (c.f., Figure 3), they either made a saccade (active shift of the stimulus across the retina) or the stimulus jumped to another location (passive shift). Trial duration was 20 seconds and there were two different trial types. In the saccade trials (Figure 5A), the stimulus was presented at the center of the screen, and the subject watched the larger of two red fixation crosses (sized 0.46° and 0.23°) located at the edge of the stimulus. After a random period of 13–16 seconds (i.e., in the sustained rivalry phase), the fixation cross shrunk. This was the cue for the subject to make a saccade to the other fixation cross located at the opposite edge of the stimulus. In stimulus jump trials, the subject fixated a green fixation cross in the center of the screen and the stimulus was located either to the left or to the right of it (Figure 5B). One second after a warning (shrinking of the fixation cross) the stimulus jumped to the other side of the fixation cross, while the subject maintained fixation. Because of the fixed delay between the warning and the actual stimulus jump, subjects could anticipate the upcoming stimulus jump, just as they could anticipate their own saccadic eye movement. Subjects could identify the trial type from the location and color of the fixation cross.

**Figure 6:**
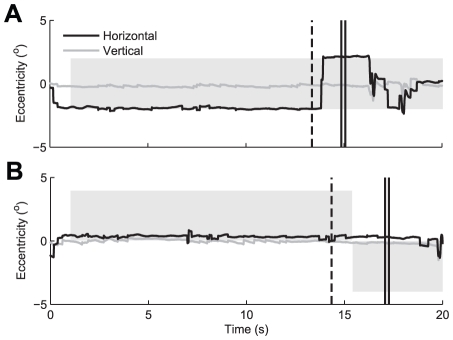
Example of eye movements. A: Saccade trial, B: stimulus jump trial. Horizontal (black) and vertical (gray) eye movements (here measured with a search coil) are shown as function of time. The gray bar indicates the horizontal position of the stimulus. The black dashed lines show the moment of the cue for the saccade or the stimulus jump, respectively. Note that the saccade and the stimulus jump result in the same retinal displacement. The double black line shows the moment the subject responded with two button presses indicating the dominance state before and after the shift, respectively. After this response, fixation was no longer required. Data from subject JG.

After the retinal image shift (either due to a saccade or a stimulus jump) the subject indicated state changes in his/her percept at the moment of retinal image shift by pressing two buttons in succession (see Figure 6, for illustration). The first button press indicated the percept dominance state immediately before the saccade or stimulus jump. The second button press indicated the percept dominance state immediately after the saccade or stimulus jump. Saccade trials and stimulus jump trials were alternated. Each subject completed a total of 200 trials per stimulus type. Saccades and stimulus jumps will together be referred to as retinal image shifts or shifts for short.

From the button-press data we calculated the probabilities of left and right eye dominance before and after the shifts, as well as the probability to switch between perceptual states or to maintain dominance at the moment of the shift. To compare the data from subjects with different individual eye/hemifield preferences, all calculations were done separately for trials in which the stimulus was presented in the left and the right hemifield. In addition, we calculated separate values for trials in which either the right eye or the left eye was dominant before the shift. This resulted in four data points per subject per stimulus type.

The resulting data were analyzed using generalized linear model regression. Towards that end, the probabilities for right eye dominance, *r = P(image in right eye perceived dominant)*, were log-transformed using the canonical link function for binomial data:
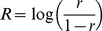
(3)Note that this new variable R represents the log-odds of right eye dominance.

The right eye dominance after a retinal image shift as a function of the right eye dominance at stimulus onset was quantified with the following regression equation:
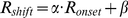
(4)The coefficients α and β as well as the corresponding t-statistics were estimated with a generalized linear model regression routine implemented in Matlab (version 7.9; glmfit). Using the same procedure, we quantified the right eye dominance after a saccade as a function of the right eye dominance after a stimulus jump:
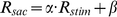
(5)Interpretation of the results, however, was easier when we plotted the difference between right eye dominance after a saccade and stimulus conditions as a function of right eye dominance after a stimulus jump. The regression lines quantifying this difference as function of right eye dominance at stimulus onset were therefore given by:
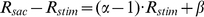
(6)Note that *Rsac – Rstim* in Equation 6 represents the log-odds ratio of right eye dominance in the saccade versus stimulus jump condition, i.e.:
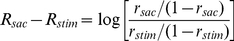
(7)


#### Results

Figure 6 illustrates the typical pattern of eye movements as observed in a saccade trial (6A) and in a stimulus jump trial (6B). These control measurements indicated that subjects maintained fixation on the fixation cross for the duration of the trial and that they made saccades of the required amplitude and direction. Saccades and stimulus jumps thus produced nearly identical retinal image shifts. Mean (±SD) reaction time of the saccades with respect to the shrinking of the fixation point (which cued the subject to make a saccade to the opposite side of the stimulus) ranged between 0.57 (±0.18) and 1.05 (±0.53) seconds.

**Figure 7:**
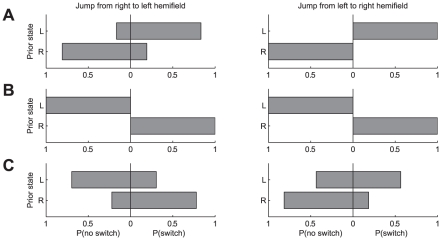
Three examples of the switch probability after stimulus jumps. Data is displayed separately for trials with left and right dominance before the shift, respectively. Left-hand panels show jumps from the right to the left hemifield and right-hand panels show jumps from the left to the right hemifield. Bars show the probability of a percept switch, P(switch) or no percept switch, P(no switch) by their position relative to zero, separately for trials in which the left eye (upper bars) or the right eye (lower bars) was dominant before the shift. Data from the face/house stimulus. A: subject FW. B: subject JV. C: subject TG.

As we will demonstrate below, retinal image shifts in the saccade versus stimulus jump conditions resulted in systematic differences. It appeared, however, that these differences could not be expressed as a simple increase or decrease in switch probability. Under both shift conditions, the probability to switch between percepts varied widely among subjects, and in addition depended strongly on the subjects' initial dominance state (i.e., just before the retinal image shift) and on the direction of the retinal image shift. Interestingly, however, we found that this seemingly idiosyncratic behavior of our subjects was systematically related to their eye/hemifield preference at stimulus onset. To illustrate this finding, Figure 7 shows the response patterns of three different subjects in the stimulus jump condition (face/house stimuli). As inferred from the response patterns in Experiment 1, one of the subjects had a systematic onset preference for stimuli in the right eye (Figure 7A, subject FW), one for stimuli in the left eye (Figure 7B, subject JV), and one for stimuli in the nasal hemifield (Figure 7C, subject TG). Note that the subject with a right eye preference at stimulus onset (Figure 7A) showed a high probability to switch to the right eye dominance state when the left eye was dominant before the jump (as shown by the upper bar pointing far rightward, P(switch) close to one), but when the right eye was dominant before the jump the probability to switch to the left eye dominance state was low (as shown by the bottom bar pointing leftward, P(no switch) close to one). This behavior was observed regardless of the direction of the stimulus jump. Choice probabilities in subject JV were also qualitatively similar for the two jump directions, but for this ‘left eye subject’ there was a high probability to switch to the left eye dominance state when the right eye was dominant before the shift, and vice versa (Figure 7B). In the ‘nasal subject’, however, the response patterns for the two jump directions were almost opposite (Figure 7C).

**Figure 8:**
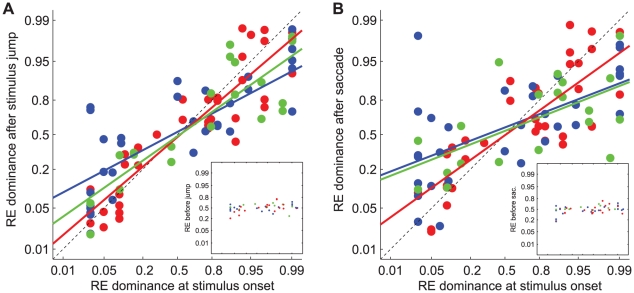
Right eye dominance after a retinal image shift versus right eye dominance at stimulus onset. A: stimulus jump trials, B: saccade trials. Blue: motion (9 subjects), red: face/house (10 subjects), green: grating (5 subjects). Data are shown on a logit scale. Note that four data points have been drawn for each subject to account for the effects of prior state and shift direction as shown in Figure 7. Insets in A and B show that *before* the retinal image shifts, the right eye was dominant in about 50% of the trials, regardless of the subjects' eye/hemifield preferences at stimulus onset.

Inspection of the raw data thus suggested that percept dominance states after a retinal image shift are systematically related to the subjects' eye/hemifield preferences. To quantify this relationship we subdivided the datasets from each subject into four subsets according to initial state and direction of the image shift, and for each subset we quantified the proportion of trials in which the right eye was dominant after the shift. We then plotted for all subjects the resulting proportions against the proportion of trials in which their first percept corresponded with the right eye (data from Experiment 1). We used logit axes for ordinate and abscissa because this is appropriate for binomial variables. Figure 8A shows the results for stimulus jump trials and Figure 8B for saccade trials. Blue, red and green symbols denote the results for the motion, face/house and grating stimuli, respectively. Note that there was a robust correlation between the probability of right eye dominance at stimulus onset and the probability of right eye dominance after a retinal image shift. Pearson's correlation coefficients for the different stimulus types ranged from 0.82 to 0.91 for the stimulus jump condition and from 0.62 to 0.83 for the saccade condition (t-tests, p<0.01). By contrast, dominance probabilities just before the shift were completely unrelated to the subjects' onset preferences. Instead they maintained a roughly-constant level of about 0.5, indicating that the shifts indeed occurred in the sustained rivalry phase. This is shown for the saccade and stimulus jump condition in the insets of Figure 8A and 8B, respectively.

Solid lines in Figure 8 are generalized linear model fits to the pooled data from all subjects (Equation 4). Note that the slopes of these regression lines are systematically different for the two shift conditions; they are lower in the saccade condition for each stimulus type. This latter observation indicates that the differences between the saccade and stimulus jump conditions also depend systematically on the subjects' eye/hemifield preferences. A direct comparison between the saccade and stimulus jump conditions thus required an analysis procedure which accounted for these preferences. We therefore split our datasets into four subsets according to initial dominance state and direction of the image shift (as in Figure 8), and we plotted the difference in right eye dominance after saccades and stimulus jumps as a function of the right eye dominance after the stimulus jumps. The difference between right eye dominance after saccades and stimulus jumps was expressed as the odds ratio for right eye dominance under the two shift conditions, and plotted on a logarithmic axis.

**Figure 9:**
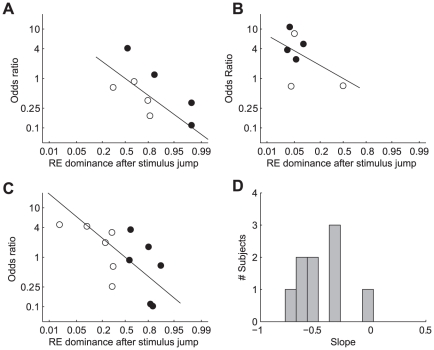
Odds ratio of eye dominance after saccades and stimulus jumps as function of onset dominance. A–C: Odds ratio of right eye dominance after saccades and stimulus jumps as function of right eye dominance after stimulus jumps, shown on a logit scale. Filled symbols: shift from left to right hemifield. Open symbols: shift from right to left hemifield. Data are from subjects FW (A) JB (B) and TG (C). D: Histogram of the regression slopes for all subjects that completed the experiment with two or three stimulus types (n = 9).

Figures 9A–C illustrate this comparison for three individual subjects (FW, JB and TG) by plotting the odds ratio of right eye dominance after saccades and stimulus jumps as function of the probability of right eye dominance after stimulus jumps. Open symbols represent data for shifts from the right to the left hemifield; filled symbols represent the data for shifts from the left to the right hemifield. Solid lines are generalized linear model fits (Equation 6) to the pooled data from the three stimulus types.

If binocular rivalry would be the same in the saccade and stimulus jump condition, the odds ratio for right eye dominance in the saccade versus stimulus jump condition would be 1 regardless of the probability of right eye dominance after stimulus jumps. In other words, all data points would lie on a horizontal line having an ordinate value of 1.0. This is clearly not observed. For the subject in Figure 9A, for example, one can see that the data points tend to fall in the bottom-right corner of the graph, which means that the subject's percepts after stimulus jumps tend to be biased towards images in the right eye (independent of the jump direction), and that the odds ratios for this subject (FW) are typically less than 1. The latter implies that, in this subject, right eye dominance after saccades is less likely than right eye dominance after stimulus jumps. For the subject in Figure 9B, on the other hand, one can see that the data points tend to fall in the top-left corner of the graph, indicating that in this subject the odds for left and right eye dominance are reversed. I.e., it appears that for this subject (JB) images in the left eye tend to dominate after stimulus jumps, and that the subject's left eye becomes less frequently dominant after saccades then after stimulus jumps. The latter follows from the fact that in this subject the odds ratios are typically larger than 1. Figure 9C illustrates the behavior of a subject with a nasal hemifield preference. Note that this subject (TG) responded as a ‘right eye subject’ for shifts to the right hemifield (filled circles) and as a ‘left eye subject’ for shift to the left hemifield (open circles).

The data thus show that the non-preferred eye/hemifield was more likely to become dominant after a saccade than after a stimulus jump. In fact, it appeared for all subjects that the odds ratios were systematically related to the eye dominance observed after stimulus jumps. More specifically, we found that if stimulus jumps resulted in a percept bias towards images in either the left or the right eye, this bias was typically reduced in the corresponding saccade condition. In our analysis, this attenuation is indexed by the negative slope of the regression lines (solid lines in Figures 9A–C). Figure 9D shows a histogram of the slopes for all subjects that completed the experiment with at least two stimulus types. For 8 out of 9 subjects, the slope was significantly below zero (t-tests, p<0.05). Offsets of the regression lines (not shown) were not significantly different from zero (t-tests, p>0.05).

**Figure 10:**
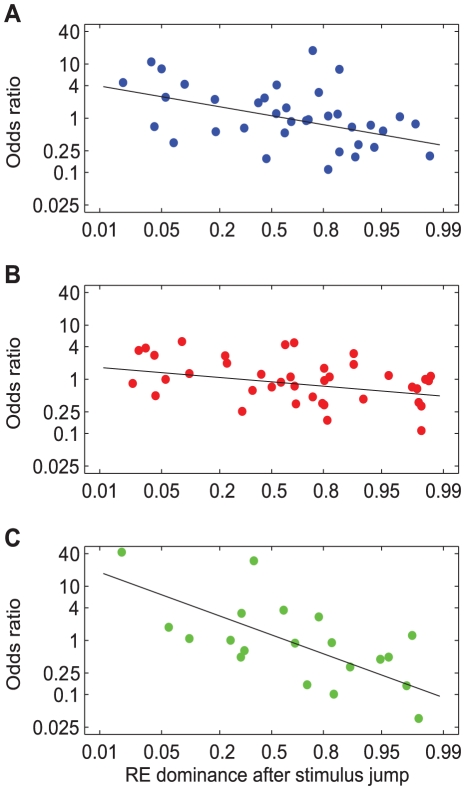
Eye dominance after retinal shift as function of onset dominance for different stimulus types. Odds ratio of right eye dominance after saccades and stimulus jumps as function of probability of right eye dominance after stimulus jumps, shown on a logit scale. A: Motion (9 subjects). B: Face/house (10 subjects). C: Gratings (5 subjects).

Figure 10 shows the same analysis as Figure 9, but now separately for the three stimulus types and pooled over all subjects. The regression lines fitted to these data had a negative slope that was significantly different from zero for all three stimulus types (t-tests, p<0.01). Slopes (mean±SE) were −0.28±0.05 for motion (9 subjects), −0.13±0.06 for face/house (10 subjects) and −0.58±0.05 for gratings (5 subjects). The differences between the face/house and the motion stimuli were not statistically significant (t-test, p>0.2). However, the effect of saccades on the rivalry bias was significantly stronger for the grating stimulus than for the motion and the face/house stimulus (t-tests, p<0.001).

## Discussion

We have examined binocular onset rivalry and the effect of saccades and stimulus jumps on perceptual state changes in 11 subjects using 3 different binocular rivalry paradigms: motion rivalry, face/house rivalry and grating rivalry. We found that the vast majority of subjects show a significant onset bias. These onset preferences are consistent across different stimulus types and experimental task conditions, but they are highly idiosyncratic across observers. Moreover, we observed a large degree of independence between rivalry at stimulus onset and that seen during sustained viewing. Our results thus corroborate and extend recent experimental findings suggesting that onset rivalry and sustained rivalry are distinct phenomena that rely on at least partly different neural mechanisms ([10], [13], [14], [24]. In addition, we found that stimuli presented in the preferred eye/hemifield are also the most likely ones to become dominant after a passive displacement of the image on the retina. In case of an active displacement (a saccade), however, this bias towards the preferred eye is significantly reduced. As we will argue below, these latter findings suggest that retinal image shifts trigger onset rivalry, and that onset rivalry depends at least partly on extra-retinal eye movement signals.

### Onset rivalry versus sustained rivalry

Previous studies have reported eye and hemifield asymmetries in switch rates and dominance durations during sustained viewing [25], [26], [27], [28]. In our experiment, we found the biggest asymmetries in eye preferences at stimulus onset and after retinal image shifts. Three subjects did not show a clear eye preference but instead had a preference for images falling on the nasal part of the retina. Fahle [29] argued that the longer dominance durations he observed for stimuli presented in the temporal hemifield (projecting onto the nasal retina) could be explained by the finding that visual hyperacuity [30], cone density [31] and cortical magnification factor [32] are higher for the nasal retina than for the temporal retina. However, all these statistics cannot readily account for the asymmetries we observed in onset rivalry, since they apply mostly to the far periphery (eccentricities >20°) while our stimuli were presented within 4° from the fovea. Ooi and He [33] suggested that a nasal hemifield preference could be useful in binocular stereovision. The mechanism they suggest might also explain the nasal preferences we found for our stimuli with small eccentricities.

Although we have tested only two locations, our results do corroborate the findings from Carter and Cavanagh [13] that strong idiosyncratic biases localized to a certain retinal location can be found at the onset of rivalry. They also support the conclusion that the onset biases disappear over time to yield a more balanced situation during sustained rivalry.

### Effect of saccades

The observation that onset rivalry differs from sustained rivalry has significant implications for rivalry in the presence of eye movements. Previous studies have shown that eye movements by themselves are not necessary to induce percept switches (e.g. [34], [35]), but for binocular rivalry, there appears to be a marked positive temporal correlation between saccades and perceptual state changes [36]. Van Dam and Van Ee [37] concluded that retinal image shifts, rather than eye movements per se cause state changes in binocular rivalry. Our current experiments shed new light on these latter results. More specifically, our finding that dominance biases at stimulus onset and after retinal image shifts are tightly correlated, strongly suggests that retinal image shifts trigger onset rivalry, and not percept switches as such.

**Figure 11:**
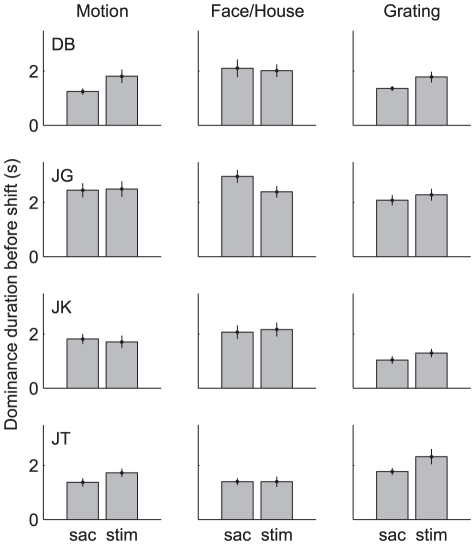
Time from last perceptual switch to the retinal image shift. Data are shown for all stimulus types (columns) and 4 subjects (rows). In each panel, the left bar show the data from saccade trials and the right bar from the stimulus jump trials. Error bars denote standard errors. Each subject completed 48–60 trials for each condition. Note the similarity of dominance durations in all subjects.

Caution is warranted though because experiments by Kanai et al. [38] suggest that changes in the fixation point, and accompanying change in attention, may elicit perceptual transitions. Thus one may wonder whether the shrinking of the fixation cross (Methods), rather than the subsequent image shifts per se, might have triggered the onset rivalry in Experiment 3. We believe, however, that this alternative interpretation is not tenable because our data show that the eye dominance states after presentation of this cue (but before the image shifts) were completely uncorrelated with the rivalry observed at stimulus onset. This was true in both the saccade and stimulus jump condition (Figure 8, insets). Another possible concern might be that dominance durations prior to the image shifts were systematically different between the saccade and stimulus jump conditions. This could be the case, for example, if subjects typically postponed their saccade until after a percept switch. To further test whether the saccade and stimulus jumps might have occurred in a systematically different phase of the (sustained) rivalry process, we therefore performed an additional set of control experiments (subjects DB, JG, JK and JT). This experiment was identical to Experiment 3 except that subjects indicated their percepts continuously by pressing one of two mouse buttons, and we measured their eye movements with an infra red eye tracker (Methods). This way, we could determine for each trial the dominance duration from the last perceptual switch until the saccade or stimulus jump. Figure 11 compares for each subject and each stimulus type the mean (±SE) duration of the dominance state prior to the saccade and stimulus jump. Note that no significant differences were found between the saccade and the stimulus condition. Taken together, we think the difference between the saccade and stimulus jump condition that we found in Experiment 3 is indeed due to the saccade itself and not to any systematic difference in prior state.

Note that the behavior that we observed after retinal image shifts is clearly different from the behavior that has been obtained in experiments with intermittent stimulus presentations at the same location. In the latter experiments (e.g., [5], [14]), short removal periods of the stimulus (<0.5 s) resulted in a high switch probability, independent of the prior state. With retinal image shifts, on the other hand, the switch probabilities strongly depend on the prior state, and on the new stimulus location. The observation that rivalry behavior after retinal shifts is strongly correlated with onset rivalry supports the notion that newly stimulated cells after a retinal shift have a different adaptation state than the cells stimulated before the switch (Introduction).

Interestingly, however, the perceptual consequence of passive, stimulus-induced image shifts was not the same as that of active, saccade-induced shifts. Indeed, saccadic eye movements not only shift the image on the retina. They also produce transient visual suppression [39], dynamic shifts of attention [40], [41] and visual receptive fields [42], [43], [44]. In view of these phenomena there are at least two possible reasons why passive versus active shifts might be different: first, during normal vision, the visuomotor system ensures that we have a stable perception of visual space despite intervening saccades (See, e.g., [45] for a review). Ross & Ma-Wyatt [19] suggested that saccades play an important role in actively maintaining perceptual continuity.

One could argue therefore that the system tries to maintain the same percept after saccades as this supports perceptual stability. If true, this would predict reduced switch probabilities in the saccade condition compared with the stimulus jump condition. On the other hand, saccades are a normal part of visual search behavior (e.g., [46], [47]) so one could also argue that redirecting the eyes to a new location in the visual field should emphasize on the gathering of new information, optimally using the inputs from both eyes. This latter notion would instead predict enhanced switch probabilities in the saccade condition.

Clearly, neither of these two interpretations can account for our results; compared with stimulus jumps, saccades produced both increases and decreases in switch probabilities depending on the preceding eye dominance state, the direction of the image shifts, and last but not least, on subject-specific biases. Even so, we did find very systematic differences between the saccade and stimulus jump conditions except that these differences were not reflected in the transition probabilities. What we found instead is that saccades consistently attenuated the subject-specific eye dominance biases after the image shifts.

**Figure 12:**
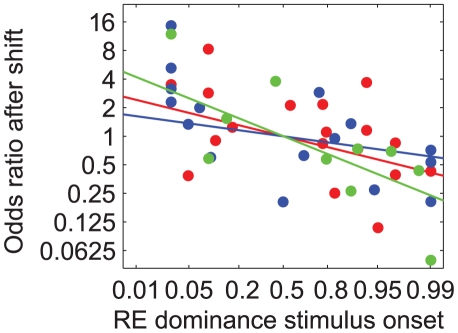
Right eye dominance after shifts as function of right eye dominance at stimulus onset. Data are shown on a logit scale with logistic regression lines. Blue: motion stimulus. Red: face/house stimulus. Green: grating stimulus.

It is unlikely that this attenuation is merely due to differences in attentional expectation because the retinal image shifts could be anticipated under both conditions (Methods). In fact, our data show that the influence of saccades is strongly correlated with the magnitude of the subjects' onset rivalry biases. The latter is shown in Figure 12 where we plot the odds ratios of right eye dominance after saccades and stimulus jumps (data from Experiment 3) as a function of right eye dominance at stimulus onset (data from Experiment 1). Correlation coefficients were −0.64, −0.52 and −0.76 for the motion, face/house and grating stimuli, respectively. We thus conclude that the influence of saccades can be understood from a systematic attenuation of the subjects' onset rivalry biases.

### Different stimulus types

The three different stimulus types that we have used in our experiments are thought to trigger at least partly different pathways in the brain. In line with this notion, we observed that for most subjects the dominance duration distributions recorded in Experiment 2 were significantly different for the three stimulus types (data not shown). In Experiment 1, quantitative differences were observed as well, albeit not systematic among subjects. Nevertheless, for all three stimulus types we observed the same remarkable dissociation between onset and sustained rivalry (Experiment 2), and a very similar influence of saccades on rivalry biases (Experiment 3).

Figure 10 suggests that the effect of saccades on the rivalry bias was significantly stronger for grating stimuli than for motion and face/house stimuli, but this difference may have resulted from pooling the data across different (numbers of) subjects, each having different onset preferences. The analysis in Figure 12 indeed demonstrates that the effect of saccades becomes indistinguishable between the three stimulus types if one accounts for the subjects' onset rivalry biases.

In a recent theoretical study, Klink et al. [14] have suggested that top-down control over bistable stimuli could interact with low-level mechanisms of adaptation at the early stages of sensory processing before perceptual conflicts are resolved and perceptual choices about bistable stimuli are made. Such an active neural control mechanism acting at lower levels could account for the fact that our results were very similar across stimuli that engage different pathways in the brain.

### Conclusions

Our results indicate that there is a large degree of independence between rivalry at stimulus onset and that seen during sustained viewing. This corroborates the hypothesis that onset rivalry and sustained rivalry are distinct phenomena that rely on at least partly different neural mechanisms. Conversely, rivalry at stimulus onset and rivalry after retinal image shifts are tightly correlated, suggesting that retinal image shifts such as those induced by saccades trigger onset rivalry. However, comparing rivalry during passive versus active retinal shifts revealed that saccades do counteract a purely retinally-driven onset rivalry in favor of perception of the image in the non-dominant eye. Our results thus indicate that non-visual signals have a significant impact on the (onset) rivalry process. Existing models of binocular rivalry need to be revised to account for these phenomena.
